# A competing risk-based prognostic model for cancer-specific survival in non-metastatic head and neck adenoid cystic carcinoma

**DOI:** 10.3389/fonc.2026.1752964

**Published:** 2026-03-23

**Authors:** Xingyuan Chen, Ruiyu Liu, Yunfan Wu, Zhuofan Wang, Rong Qiu, Juan Li, Yuxiang Wang

**Affiliations:** 1 Department of Radiation Oncology, The Fourth Hospital of Hebei Medical University & Hebei Clinical Research Center for Radiation Oncology, Shijiazhuang, China; 2 Department of Oncology, Chengdu Women’s and Children’s Central Hospital, School of Medicine, University of Electronic Science and Technology of China, Chengdu, China

**Keywords:** cancer-specific survival, competing risks model, fine–gray model, head and neck adenoid cystic carcinoma, prognostic nomogram

## Abstract

**Introduction:**

Traditional Cox regression may yield biased estimates when competing events are present, limiting the accuracy of prognostic analyses in head and neck adenoid cystic carcinoma (HNACC). This study applied the Fine–Gray competing risks model to identify independent prognostic factors associated with HNACC-related mortality and develop a predictive nomogram using data from the Surveillance, Epidemiology, and End Results (SEER) database.

**Methods:**

Patients diagnosed with HNACC between 2004 and 2015 were identified from the SEER database. Univariable analyses were performed using Gray’s test and the cumulative incidence function, while multivariable analyses employed Cox regression and Fine–Gray proportional subdistribution hazards models. A nomogram was developed to predict 3-, 5-, and 10-year cancer-specific survival (CSS) and validated in an independent cohort.

**Results:**

A total of 2,688 eligible patients were included. During follow-up, 1,046 deaths occurred, of which 673 were attributable to HNACC. The Fine–Gray model identified age, T-stage, N-stage, POCRT status, PORT status, and perineural invasion (PNI) as independent prognostic factors for CSS. These variables were incorporated into a nomogram that demonstrated excellent discrimination, with concordance indices of 0.818, 0.806, and 0.822 for 3-, 5-, and 10-year predictions in the training cohort, and 0.909, 0.931, and 0.965, respectively, in the validation cohort.

**Conclusions:**

The competing risks model identified key prognostic factors influencing CSS in HNACC. The derived nomogram provides individualized survival estimates, offering a practical tool to support clinical decision-making.

## 1 Introduction

Adenoid cystic carcinoma (ACC) is characterized by slow but infiltrative growth and a high propensity for perineural invasion (PNI). The 5-year overall survival rate ranges from 68% to 90%, whereas long-term outcomes worsen substantially, with 10- and 15-year survival rates falling to 52% and 28%, respectively. These trends highlight the distinct biological behavior of ACC compared with other salivary gland tumors (1, 2). Head and neck ACC (HNACC) is particularly prone to PNI and intracranial extension because it frequently arises near critical anatomical structures, making the achievement of clear surgical margins challenging. Current management primarily involves surgical resection with negative margins followed by adjuvant radiotherapy. The role of multimodal therapy for HNACC remains a subject of debate (1–4). Therefore, a comprehensive understanding of its clinical characteristics, imaging techniques, and pathology is essential to establish effective guidelines for diagnosis, preoperative evaluation, and treatment planning.

Previous prognostic studies have largely employed conventional statistical approaches such as Kaplan–Meier estimation and Cox proportional hazards regression. These models do not adequately account for competing risks, such as non-cancer deaths (1, 3, 4). Because such deaths compete with cancer-related outcomes, analyses that ignore them may provide biased estimates of prognostic factors. When assessing prognostic variables in cancer patients, it is crucial to consider non-cancer-related deaths as competing events and focus specifically on cancer-related mortality. To date, no studies have systematically examined prognostic factors for invasive HNACC subtypes using a competing risks framework.

Competing risks data are common in oncology and arise across virtually all cancer types (5–7). Standard Cox regression models fail do not account for competing risks, which can lead to an overestimation of the incidence of favorable events and to inaccurate risk stratification. Although using all-cause mortality as the study endpoint avoids this bias, it obscures the specific effects of prognostic factors on cancer-related mortality (5, 7). For such data, cumulative incidence curves are recommended because they provide a clearer understanding of the frequency, timing, and interdependence of competing events. In the analysis of terminal events, any event other than the primary event of interest is typically treated as a competing event. For non-terminal events, such as chronic diseases or infections, death before the occurrence of the event of interest generally serves as the competing event (5). The Fine–Gray proportional subdistribution hazards model is regarded as the most appropriate method for estimating survival probabilities in the presence of competing risks (7). The cumulative incidence function (CIF) assumes that only one event occurs at a given time and maintains the property that the sum of CIFs for all categories equals the CIF for the composite event (5). Accordingly, in the present study, we calculated the CIF for cancer-specific death and used the Fine–Gray model to identify prognostic factors for HNACC.

The Surveillance, Epidemiology, and End Results (SEER) program, established by the National Cancer Institute, maintains a comprehensive registry covering 28% of the U.S. population and includes cancer data from 18 geographical regions. Because SEER data are publicly available, informed consent are not required for their use. Additionally, we obtained a license to access SEER chemotherapy data and permission to use SEER*Stat software for analysis (8). The objective of this study was to identify prognostic factors associated with HNACC using a competing risks model and to develop a nomogram for predicting 3-, 5-, and 10-year cancer-specific survival (CSS).

## 2 Methods

### 2.1 Data collection

Patients diagnosed with HNACC between 2004 and 2015 were identified from the SEER database using the ICD-O-3 morphology code “8200/3”. The inclusion criteria were as follows: (1) histologically confirmed ACC; (2) treatment with radical surgery; and (3) availability of tumor staging data according to the AJCC 7th edition. The exclusion criteria were: (1) absence of surgical treatment or unknown surgical status; (2) duplicate patient identifiers with inconsistent information; (3) missing TNM stage or sex data; (4) receipt of chemotherapy before surgery; and (5) presence of distant metastasis at diagnosis.

The following variables were collected: age at diagnosis, sex, race, primary tumor site, laterality, lymph node dissection (LND) status, T and N stage, PNI, and treatment modality (including postoperative radiotherapy [PORT] and postoperative chemoradiotherapy [POCRT]). Patients who received overlapping PORT and POCRT were strictly excluded during data screening to ensure mutual exclusivity of these variables. The patient selection process is illustrated in **Supplementary Figure 1**. In addition, an external validation cohort was established using clinical data from patients with HNACC treated at the Fourth Hospital of Hebei Medical University between January 2010 and December 2020. This study was approved by the ethics committee of the Fourth Hospital of Hebei Medical University (2022KY262).

### 2.2 Statistical analysis

A total of 2688 eligible patients were randomly divided into training and validation sets in a 7:3 ratio using R software (version 3.5.3). Baseline characteristics of both cohorts were summarized, and differences between groups were assessed using the Student’s *t*-test for continuous variables and the χ^2^ test for categorical variables. Optimal cut-off values for continuous variables such as tumor size and age were determined using the X-tile software, and these variables were subsequently converted into categorical form.

To identify clinical variables significantly associated with cancer-specific survival (CSS), we employed the Least Absolute Shrinkage and Selection Operator (LASSO) method for variable selection, followed by Cox regression analysis for model development and visualization (9, 10). The optimal regularization parameter (λ) in the LASSO model was determined through generalized cross-validation, and model performance was assessed by evaluating changes in the area under the curve (AUC) across different log(λ) values (11).

Finally, a proportional subdistribution hazards model (Fine–Gray model) was applied to account for competing risks, and a nomogram was developed to predict 3−, 5−, and 10−year CSS. The predictive accuracy of the model performance was quantified by the concordance index (C-index), and its agreement between predicted and observed outcomes was examined using calibration curves in the training and validation cohorts. The specific R packages used for the key analyses were as follows: the cmprsk package (v2.2-11) for Fine–Gray model fitting; the rms package (v6.7-1) for nomogram construction, C-index calculation, and calibration curve generation; the glmnet package (v4.1-8) for LASSO regression; and the survminer package (v0.4.9) for CIF curve visualization.

### 2.3 Definition of outcomes

Cancer-specific survival (CSS) was defined as the time from the date of curative surgery to the date of death specifically attributed to HNACC, as determined by the SEER Cause of Death–Recode variable, ICD-O-3 code 8200/3. Patients who died from other causes or were lost to follow-up were censored at the date of last contact or the study cutoff date.

Other-cause death (OCD) was defined as death due to any cause other than HNACC, such as cardiovascular disease, other malignancies, or infectious diseases, according to the Cause of Death–Recode variable. In the Fine–Gray competing risk analysis, OCD was treated as a competing event and incorporated into the subdistribution hazard model.

## 3 Results

### 3.1 Patient characteristics

The baseline characteristics of the 2688 patients, including 1883 (70%) in the training cohort and 805 (30%) in the validation cohort, are summarized in **Table 1**. The median age in both cohorts was 60 years. All baseline variables were well-balanced between the two groups.

**Table 1:** Basic characteristics of patients in this study.

Variable	Category	Training cohort (n)	Validation cohort (n)	External validation cohort (n)	*P-value*
Gender	Female	1120	452	80	0.1102256
	Male	764	353	71	
Age	<60	967	405	80	0.620331
	≥60	917	400	71	
Race	White	1416	609	0	0.6423584
	Black	199	89	0	
	Others	268	107	151	
POCRT	Yes	230	91	55	0.4995986
	No	1653	714	96	
PORT	Yes	1269	535	111	0.6384615
	No	614	270	40	
LND	Yes	842	349	63	0.514756
	No	1041	456	88	
T-staging	T1	514	214	21	0.819848
	T2	417	215	45	
	T3	365	133	54	
	T4	554	243	31	
N-staging	N0	1656	708	66	0.6476029
	N1	102	48	57	
	N2	96	41	8	
	N3	29	8	20	
Stage	I	526	201	20	0.7561243
	**II**	380	200	36	
	III	358	137	60	
	IV	619	267	35	
Laterality	Left	691	280	60	0.8202678
	Right	647	301	54	
	Others	541	222	37	
Primary site	Parotid	499	183	45	0.1948157
	Submandibular	390	191	54	
	NSC	312	134	13	
	oral cavity	451	179	31	
	Others	231	118	8	
PNI	Yes	375	155	69	0.3948157
	No	756	314	43	
X	Unknow	752	336	38	

LND, lymph node dissection; OCD, other-cause death; PNI, perineural invasion; POCRT, postoperative chemoradiotherapy; PORT, postoperative radiotherapy; NSC, nasal and paranasal sinus carcinoma.

Bold values indicate the statistical significance of the differences between groups, with P < 0.05 considered statistically significant.

The external validation cohort comprised 151 patients with HNACC who were treated at the Fourth Hospital of Hebei Medical University between January 2010 and December 2020.

### 3.2 Univariate analysis

**Figure 1:**
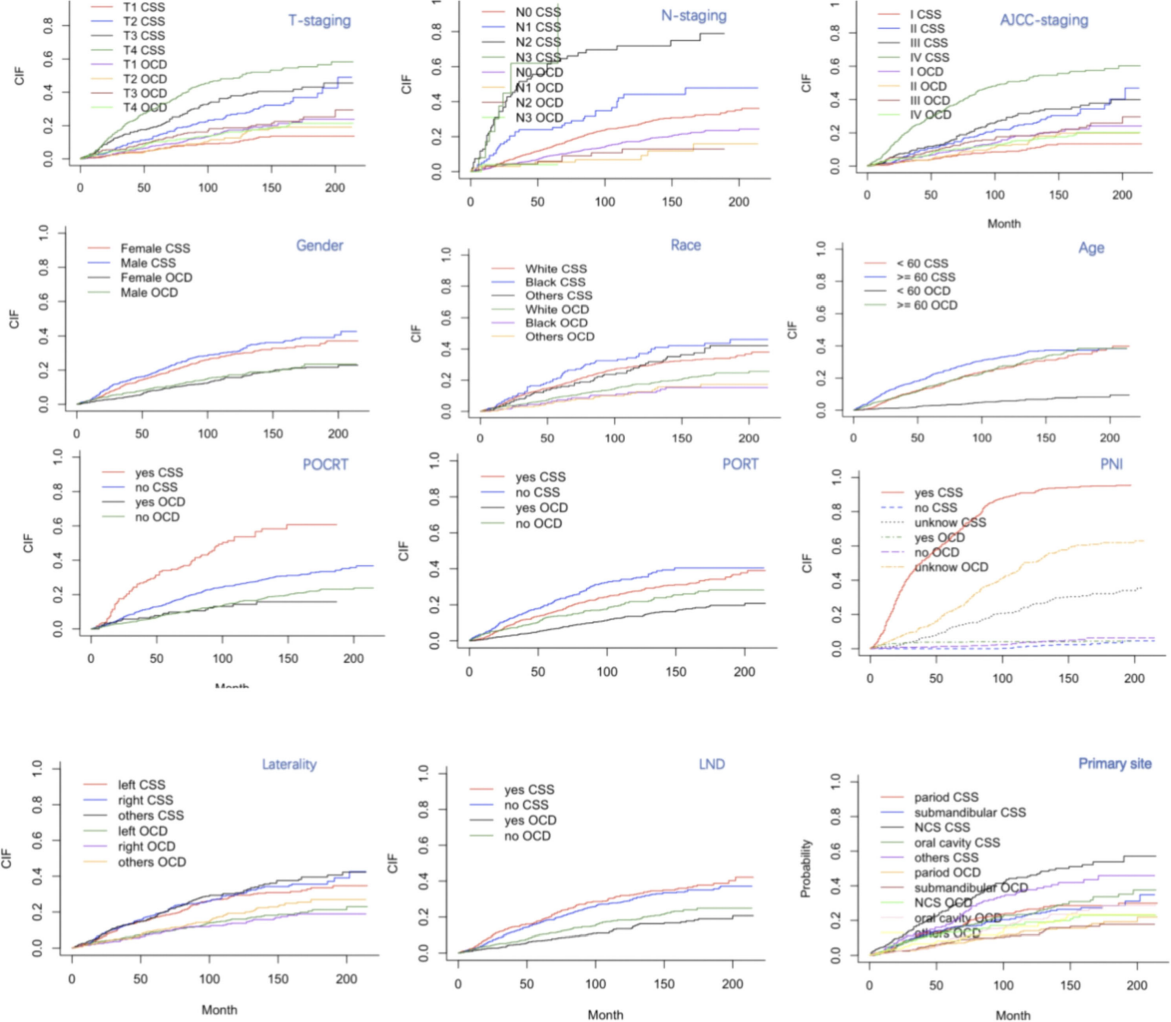
Cumulative incidence function (CIF) curves for cancer-specific survival (CSS) and other cause death (OCD) among patients with head and neck adenoid cystic carcinoma.

The 3-, 5-, and 10-year cumulative incidence rates of CSS and other-cause death (OCD) were calculated. Age, T-stage, N-stage, AJCC stage, PNI, primary tumor site, PORT, and POCRT were significantly associated with both outcomes (*p* < 0.05). Laterality, race, sex, and lymph node dissection (LND) status showed no significant association (*p* > 0.05). The corresponding CIF curves are presented in **Figure 1**, and the comparative results of CSS and OCD are presented in **Table 2**.

**Table 2:** The cumulative incidence function (CIF) of CSS and OCD among patients with head and neck adenoid cystic carcinoma (HNACC).

Variables	Category	N	CSS				OCD			
			1-year (95% CI)	3-year (95% CI)	5-year (95% CI)	*p*	1-year (95% CI)	3-year (95% CI)	5-year (95% CI)	*P-value*
Race	White	1,422	3.3% (2.4%, 4.3%)	12% (9.8%, 13%)	17% (15%, 19%)	0.42	2.0% (1.4%, 2.9%)	5.2% (4.1%, 6.5%)	8.3% (6.9%, 10%)	0.38
	Black	199	5.3% (2.7%, 9.2%)	15% (10%, 21%)	21% (15%, 28%)		1.5% (0.42%, 4.2%)	6.8% (3.7%, 11%)	8.3% (4.7%, 13%)	
	Others	263	3.6% (1.8%, 6.5%)	12% (8.1%, 17%)	16% (11%, 21%)		1.2% (0.33%, 3.2%)	3.9% (1.9%, 7.0%)	7.4% (4.3%, 12%)	
Age	<60	968	1.7% (1.0%, 2.7%)	9.4% (7.5%, 11%)	14% (12%, 16%)	<0.001	0.75% (0.34%, 1.5%)	1.6% (0.91%, 2.6%)	2.7% (1.7%, 4.0%)	<0.001
	≥60	916	6.5% (5.0%, 8.2%)	16% (14%, 19%)	22% (19%, 25%)		3.1% (2.1%, 4.4%)	8.6% (6.8%, 11%)	14% (12%, 17%)	
T	T1	514	1.1% (0.48%, 2.4%)	3.4% (2.1%, 5.3%)	5.1% (3.3%, 7.3%)	<0.001	0.95% (0.37%, 2.1%)	4.5% (2.9%, 6.6%)	6.4% (4.4%, 8.8%)	0.1
	T2	417	2.4% (1.2%, 4.2%)	6.9% (4.7%, 9.7%)	12% (9.0%, 16%)		1.4% (0.59%, 2.9%)	3.8% (2.2%, 6.1%)	6.3% (4.1%, 9.1%)	
	T3	365	5.1% (3.1%, 7.9%)	17% (13%, 21%)	23% (18%, 28%)		3.9% (2.2%, 6.4%)	7.2% (4.7%, 10%)	11% (7.9%, 15%)	
	T4	587	7.5% (5.4%, 9.9%)	23% (20%, 27%)	32% (28%, 37%)		1.9% (0.96%, 3.3%)	5.0% (3.3%, 7.1%)	9.4% (6.9%, 12%)	
N	N0	1656	2.8% (2.1%, 3.7%)	9.0% (7.6%, 10%)	14% (12%, 16%)	<0.001	1.8% (1.2%, 2.6%)	5.1% (4.1%, 6.3%)	8.4% (7.0%, 10%)	0.083
	N1	102	6.3% (2.6%, 12%)	24% (16%, 34%)	26% (17%, 35%)		1.0% (0.09%, 5.0%)	2.1% (0.40%, 6.7%)	4.6% (1.5%, 11%)	
	N2	96	17% (10%, 26%)	52% (40%, 62%)	62% (50%, 72%)		3.3% (0.87%, 8.5%)	5.6% (2.1%, 12%)	7.1% (2.8%, 14%)	
	N3	29	24% (9.6%, 43%)	75% (12%, 96%)	75% (12%, 96%)		4.0% (0.27%, 17%)	4.0% (0.27%, 17%)	4.0% (0.27%, 17%)	
POCRT	yes	225	8.8% (5.5%, 13%)	29% (23%, 36%)	37% (30%, 44%)	<0.001	3.7% (1.7%, 6.9%)	5.8% (3.2%, 9.7%)	9.3% (5.6%, 14%)	0.85
	no	1,658	3.4% (2.6%, 4.4%)	10% (8.9%, 12%)	15% (14%, 17%)		1.6% (1.1%, 2.3%)	4.9% (3.9%, 6.1%)	8.0% (6.6%, 9.5%)	
PORT	yes	1,271	3.0% (2.2%, 4.1%)	11% (9.3%, 13%)	16% (14%, 18%)	0.002	0.82% (0.42%, 1.5%)	3.3% (2.3%, 4.4%)	6.0% (4.6%, 7.5%)	<0.001
	no	612	6.1% (4.4%, 8.2%)	16% (13%, 19%)	22% (19%, 26%)		4.1% (2.7%, 5.9%)	8.6% (6.4%, 11%)	13% (9.9%, 16%)	
LND	yes	838	4.0% (2.8%, 5.6%)	13% (11%, 16%)	18% (15%, 21%)	0.54	1.5% (0.82%, 2.5%)	4.7% (3.3%, 6.4%)	7.2% (5.4%, 9.4%)	0.12
	no	1,045	4.0% (3.0%, 5.4%)	12% (10%, 14%)	18% (15%, 20%)		2.2% (1.4%, 3.2%)	5.3% (4.0%, 6.8%)	8.8% (7.1%, 11%)	
Stage	I	526	0.99% (0.38%, 2.2%)	2.5% (1.4%, 4.2%)	4.2% (2.6%, 6.4%)	<0.001	1.0% (0.38%, 2.2%)	4.7% (3.0%, 6.9%)	6.7% (4.6%, 9.2%)	0.42
	**II**	380	1.6% (0.66%, 3.3%)	5.7% (3.6%, 8.5%)	10% (7.4%, 14%)		1.3% (0.50%, 2.9%)	4.0% (2.3%, 6.4%)	6.7% (4.3%, 9.7%)	
	III	358	2.7% (1.3%, 4.9%)	12% (8.3%, 15%)	16% (12%, 21%)		3.3% (1.7%, 5.6%)	6.5% (4.2%, 9.6%)	11% (7.3%, 14%)	
	IV	619	8.9% (6.8%, 11%)	26% (23%, 30%)	35% (31%, 40%)		2.2% (1.2%, 3.6%)	5.0% (3.4%, 7.0%)	9.0% (6.7%, 12%)	
Laterality	left	691	3.7% (2.4%, 5.4%)	12% (9.8%, 15%)	18% (15%, 21%)	0.62	2.2% (1.2%, 3.5%)	5.2% (3.7%, 7.2%)	8.7% (6.5%, 11%)	0.61
	right	647	3.7% (2.4%, 5.4%)	13% (10%, 15%)	18% (15%, 21%)		1.7% (0.90%, 2.9%)	4.7% (3.2%, 6.6%)	6.9% (5.0%, 9.2%)	
	others	545	4.7% (3.1%, 6.8%)	13% (9.8%, 16%)	17% (14%, 21%)		1.8% (0.88%, 3.2%)	5.2% (3.4%, 7.4%)	8.9% (6.5%, 12%)	
site	parotid	499	2.6% (1.4%, 4.3%)	11% (8.6%, 15%)	15% (11%, 18%)	<0.001	2.1% (1.1%, 3.8%)	4.8% (3.1%, 7.1%)	7.3% (5.0%, 10%)	0.64
	submandibular	390	4.6% (2.8%, 7.0%)	11% (8.1%, 15%)	15% (12%, 19%)		1.3% (0.48%, 2.8%)	3.3% (1.8%, 5.5%)	6.6% (4.3%, 9.6%)	
	NSC	312	7.4% (4.8%, 11%)	21% (16%, 26%)	29% (24%, 35%)		3.3% (1.7%, 5.7%)	7.9% (5.2%, 11%)	11% (7.3%, 15%)	
	oral cavity	451	2.2% (1.1%, 4.0%)	8.8% (6.2%, 12%)	13% (9.6%, 17%)		0.73% (0.20%, 2.0%)	2.6% (1.3%, 4.6%)	7.6% (5.1%, 11%)	
	others	231	4.7% (2.5%, 8.0%)	13% (8.9%, 18%)	21% (16%, 27%)		2.6% (1.1%, 5.2%)	8.5% (5.3%, 13%)	10% (6.5%, 14%)	
PNI	yes	375	16% (12%, 20%)	49% (44%, 54%)	64% (59%, 69%)	<0.001	2.4% (1.2%, 4.4%)	3.5% (2.0%, 5.8%)	3.8% (2.2%, 6.2%)	<0.001
	no	756	0.00% (—%, —%)	0.00% (—%, —%)	0.00% (—%, —%)		0.40% (0.11%, 1.1%)	0.93% (0.42%, 1.8%)	1.3% (0.69%, 2.4%)	
	Unknown	752	1.1% (0.54%, 2.1%)	5.1% (3.4%, 7.2%)	12% (9.0%, 15%)		3.8% (2.5%, 5.4%)	12% (9.1%, 14%)	22% (18%, 26%)	

HNACC, head and neck adenoid cystic carcinoma; PNI, perineural invasion; POCRT, postoperative chemoradiotherapy; PORT, postoperative radiotherapy; NSC, nasal and paranasal sinus carcinoma; OCD, other-cause death; CSS, cancer-specific survival.

Bold values indicate the statistical significance of the differences between groups, with P < 0.05 considered statistically significant.

### 3.3 Multivariate analysis

Lasso regression was performed for variable selection, with coefficient profiles illustrated in **Supplementary Figure 2**. Using 10-fold cross-validation, the optimal regularization parameter (λ) was identified as 0.075, producing a parsimonious model with robust predictive performance. Significant predictors (*p* < 0.05) selected by Lasso (age, PNI, PORT, POCRT, T-stage, and N-stage) were subsequently incorporated into multivariate Cox regression analysis.

To account for competing risks, we applied the Fine**–**Gray proportional sub-distribution hazards model and compared its results with those from the cause-specific hazards model. The cancer-specific hazards model indicated that age, T stage, N stage, POCRT, PORT, and PNI were independent prognostic factors for HNACC (*p* < 0.05). The Fine-Gray model confirmed these findings, identifying the following as significant prognostic factors (**Table 3**): POCRT status (HR = 0.60 for no, 95% CI 0.47–0.78), PORT status (HR = 1.38 for no, 95% CI 1.12–1.69), N stage (HR = 1.75 for N1, 95% CI 1.21–2.53; HR = 3.86 for N2, 95% CI 2.78–5.34; HR = 5.87 for N3, 95% CI 2.77–12.5), T stage (HR = 2.12 for T1, 95% CI 1.53–2.93; HR = 2.90 for T2, 95% CI 2.08–4.06; HR = 3.76 for T3, 95% CI 2.76–5.12), PNI status (HR = 0.00 for negative, 95% CI 0.00–0.01; HR = 0.07 for unknown, 95% CI 0.06–0.10), and age (HR = 1.37 for ≥60, 95% CI 1.14–1.66).

**Table 3:** Two models of prognostic factors in patients with HNACC were analyzed using multivariable analysis.

Variables	N	Fine-gray model		Cox model	
		HR*^1^* (95% CI)	*p*	HR (95% CI)	*p*
POCRT					
yes	233	reference		reference	
no	1,650	0.60 (0.47–0.78)	<0.001	0.73 (0.59–0.91)	<0.001
PORT					
yes	1,267	reference		reference	
no	616	1.38 (1.12–1.69)	0.002	1.21 (1.04–1.41)	0.01
N					
N0	1,661	reference		reference	
N1	101	1.75 (1.21–2.53)	0.003	1.22 (0.9–1.66)	0.20
N2	99	3.86 (2.78–5.34)	<0.001	1.64 (1.26–2.15)	<0.001
N3	22	5.87 (2.77–12.5)	<0.001	2.89 (1.64–5.08)	<0.001
T					
T1	533	reference		reference	
T2	438	2.12 (1.53–2.93)	<0.001	1.26 (0.99–1.61)	0.06
T3	346	2.90 (2.08–4.06)	<0.001	1.25 (0.96–1.61)	0.10
T4	566	3.76 (2.76–5.12)	<0.001	0.96 (0.76–1.22)	0.75
Primary site					
parotid	467	reference		reference	
submandibular	403	0.96 (0.73–1.27)	0.8	1.03 (0.81–1.31)	0.8021
NSC	319	1.03 (0.78–1.36)	0.9	1.1 (0.88–1.37)	0.4258
oral cavity	435	0.98 (0.74–1.29)	0.9	0.99 (0.78–1.24)	0.901
others	259	0.98 (0.72–1.31)	0.9	1.01 (0.79–1.28)	0.9432
age					
<60	960	reference		reference	
≥60	923	1.37 (1.14–1.66)	0.001	1.96 (1.67–2.29)	0.00
PNI					
yes	375	reference		reference	
no	756	0.00 (0.00–0.01)	<0.001	0.02 (0.01–0.02)	<0.001
unknown	752	0.07 (0.06–0.10)	<0.001	0.36 (0.3–0.44)	<0.001

HNACC, head and neck adenoid cystic carcinoma; PNI, perineural invasion; POCRT, postoperative chemoradiotherapy; PORT, postoperative radiotherapy; NSC, nasal and paranasal sinus carcinom.

### 3.4 Construction and verification of the nomogram

**Figure 2:**
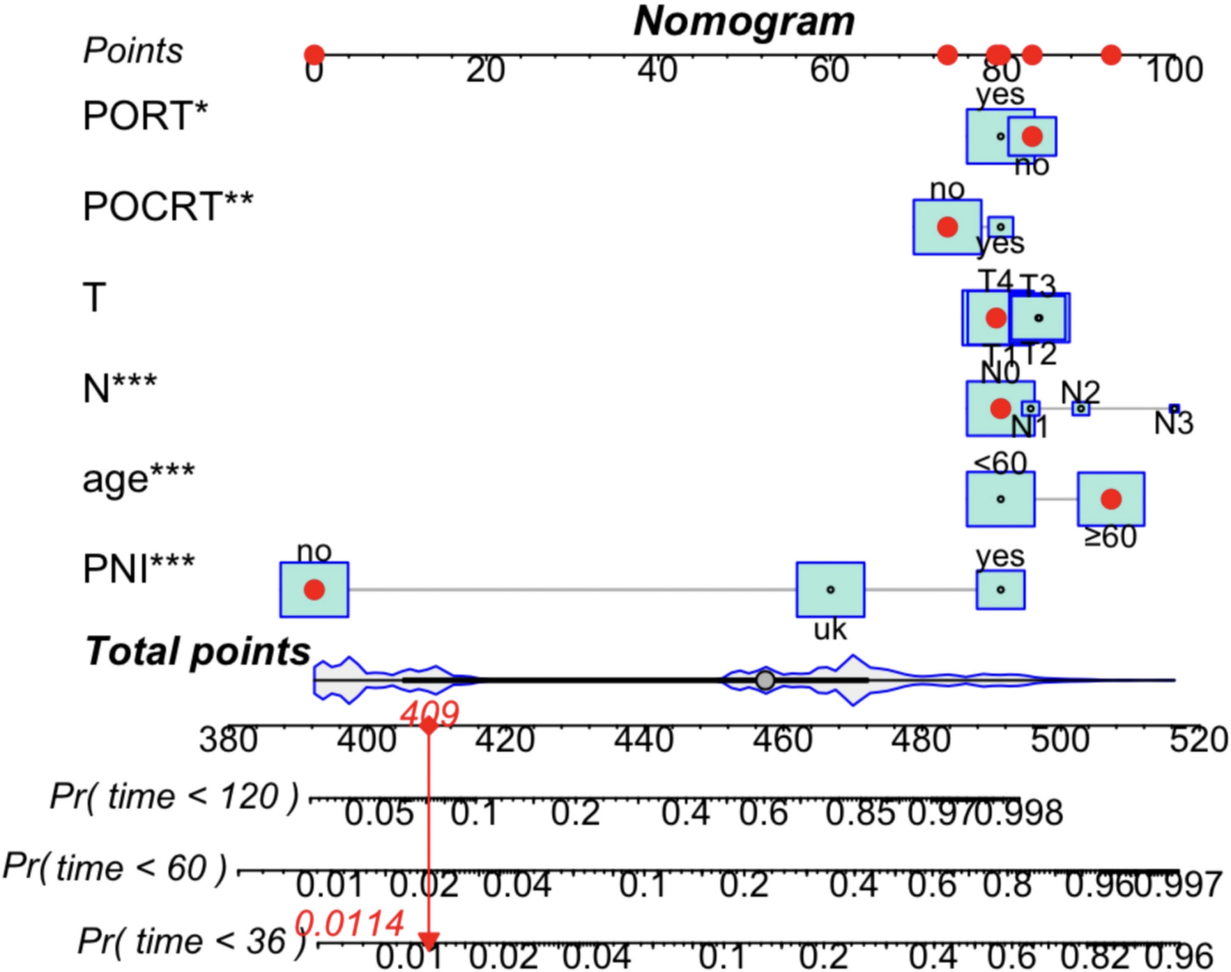
Nomogram based on the competing risks analysis for predicting 3-, 5-, and 10-year cancer-specific survival (CSS) probabilities in patients with head and neck adenoid cystic carcinoma (HNACC). Red circles: Represent the specific point value assigned to each category of the included prognostic factors (age, T-stage, N-stage, PNI status, PORT, POCRT), with the corresponding score directly readable on the vertical “Points” scale. Square sizes: Reflect the prognostic weight/relative importance of each factor for HNACC patients’ CSS; larger square areas indicate a stronger prognostic impact on cancer-specific mortality (T-stage has the largest square, as the most critical prognostic factor). Line connecting 409 to 0.0114: Denotes the quantitative correspondence between the maximum total prognostic score (409 points, the sum of the highest point values of all risk factors, representing the most unfavorable clinical status) and the corresponding 10-year CSS probability (0.0114, i.e., 1.14%). Statistical significance: *P < 0.05, **P < 0.01, ***P < 0.001.

Based on the Fine–Gray competing risks model, a nomogram incorporating six independent prognostic factors was constructed (**Figure 2**) to predict 3-year, 5-year, and 10-year cancer-specific survival in patients with non-metastatic HNACC. T-stage was identified as the most influential prognostic factor for CSS, as indicated by the largest square size in the nomogram, followed by N-stage and PNI status.

### 3.5 Calibration and discrimination

**Figure 3:**
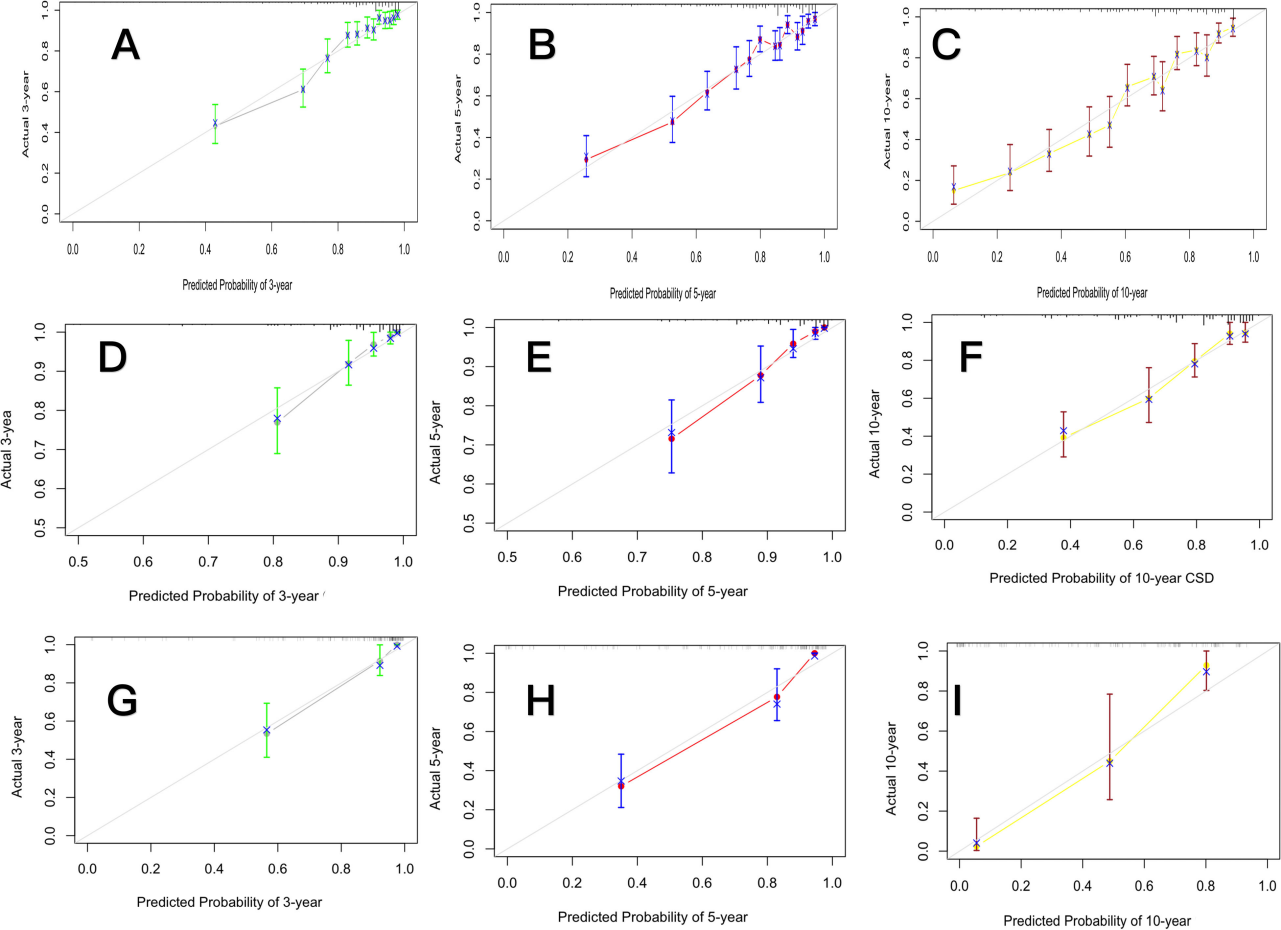
Calibration curves for the training cohort **(A–C)**, test cohort **(D–F)**, and the external validation cohort **(G–I)**, showing the agreement between predicted and observed cancer-specific survival probabilities at 3, 5, and 10 years.

The concordance index (C-index) with its 95% confidence interval (CI) was employed to quantify the discriminative ability of the Fine–Gray–based nomogram. In the training cohort, the C-index values for 3-, 5-, and 10-year CSS were 0.902 (0.885–0.919), 0.937 (0.924–0.950), and 0.964 (0.953–0.975), respectively. In the test cohort, the model exhibited robust discriminative performance, with C-index values of 0.818 (0.801–0.835), 0.806 (0.789–0.823), and 0.822 (0.805–0.839) for 3-, 5-, and 10-year CSS, respectively. In the external validation cohort, discrimination remained robust, with corresponding C-index values of 0.909 (0.892–0.926), 0.931 (0.918–0.944), and 0.965 (0.954–0.976) for 3-, 5-, and 10-year CSS, respectively (**Figure 3**).

Because the 95% CIs of the C-index values for the Fine–Gray and Cox regression models did not overlap, the difference in discriminative performance was considered statistically significant. To further support this finding, we compared the Akaike information criterion (AIC) values of the two models. The Fine–Gray competing risks model yielded an AIC of 5842.3, while the traditional Cox regression model yielded an AIC of 6019.7. The substantially lower AIC of the Fine–Gray model, with a ΔAIC greater than 100, indicates a markedly better trade-off between goodness-of-fit and model complexity.

## 4 Discussion

Previous prognostic studies of ACC and its subtypes have rarely incorporated competing risk analyses, relying instead on conventional Cox or logistic regression models. Commonly reported prognostic factors include advanced age, poor differentiation, PNI, and larger tumor size (1–4). Accurate prognostic evaluation is essential for individualized treatment planning, assessing therapeutic efficacy, and guiding clinical follow-up.

In the prospective REFCOR multicenter study, Atallah et al. (12). reported 5- and 10-year survival rates of 85% and 67%, respectively, among HNACC patients receiving postoperative radiotherapy. In our SEER-based cohort of 2688 patients, 1,046 deaths occurred, of which only 673 were cancer-related. Conventional survival methods, such as Kaplan–Meier and Cox regression, treat non-cancer deaths as censored, which may overestimate CSS and violate the model assumptions. The Fine–Gray competing risks model, in contrast, provides more accurate hazard estimation and is therefore more appropriate for identifying independent prognostic factors in HNACC.

Our results demonstrated significant differences between the Fine–Gray and Cox models. In the Fine–Gray model, T-stage emerged as a significant prognostic factor, whereas it was not an independent prognostic factor in Cox regression. Both models identified age, N-stage, PORT, POCRT, and PNI as significant variables, although the magnitude of risk estimates differed. Other variables showed no significant prognostic impact in either model.

Multiple studies have confirmed the prognostic relevance of T and N stage, although their relative importance varies (12, 13). While some authors identified both as significant predictors, Nascimento et al. (14) reported that the N stage was associated with local control but not survival. Ali et al. (15) found that pathologic T-stage independently predicted local failure and lymph node metastasis in salivary ACC. In the present study, both T and N stages were significant predictors of disease-specific survival using the Fine–Gray model. The lack of T-stage significance in the Cox model likely reflects the influence of censoring of non-cancer deaths, which can lead to underestimation of cancer-specific cumulative incidence. This underscores the advantage of the Fine–Gray approach in handling competing events. The association between T/N stage and distant metastasis could not be evaluated due to limitations of the SEER database. Although tumor grade is clinically relevant, its prognostic role remains controversial (14, 16) and was not validated in this study.

In this study, the optimal cut-off value for the continuous variable age was determined using X-tile software for survival analysis. Older age (≥60 years) was associated with increased cancer-specific mortality, consistent with reports linking advanced age to higher distant metastasis rates in early-stage ACC (17, 18). The poorer outcomes in older patients may reflect comorbidities and reduced treatment tolerance (18). Notably, risk estimates for older age differed between models: the Fine–Gray model yielded an HR of 1.37 (95% CI: 1.14–1.66), while Cox regression produced a higher HR of 1.96 (95% CI: 1.67–2.29), suggesting that conventional models may overestimate risk in the presence of competing events. While some studies have linked both younger (<40) and older (>60) age to worse survival (17), our findings did not confirm an association between age and metastatic disease. Some reports have also questioned the independent prognostic value of age in ACC (19), warranting further research.

Postoperative radiotherapy (PORT) is widely used in the management of HNACC, though its survival benefit remains debated. Several studies, including a large SEER analysis by Mahmood et al. (20) and reports by Zupancic et al. (21) have associated PORT with improved survival in high-grade or locally advanced salivary gland tumors. In our univariate analysis, both Cox regression and the Fine–Gray model identified PORT status as a significant adverse prognostic factor (*p* < 0.05). Therefore, PORT may be recommended as a treatment option.

Currently, no standardized chemotherapy regimen exists for ACC, and effective second-line options remain limited. Zupancic et al. (21) observed lower overall survival in patients receiving postoperative chemoradiotherapy (POCRT) compared to radiotherapy (PORT) alone. Similarly, Hsieh et al. (22) reported higher mortality and toxicity with combination chemotherapy relative to radiotherapy alone in salivary gland carcinomas, while Chau et al. (23) noted that ACC exhibits relative chemoresistance compared to other salivary gland malignancies. In our analysis, both Cox regression (HR = 0.73, 95% CI: 0.59–0.91) and the Fine–Gray model (HR = 0.60, 95% CI: 0.47–0.78) showed significantly lower risk without chemotherapy, though the competing risks model suggested a smaller effect size. Chemotherapy should be administered only when it provides a survival benefit, as inappropriate use may increase the risk of mortality.

HNACC typically exhibits slow growth but marked neurotropism, enabling early perineural spread and causing localized pain (24, 25). Recurrence is common and closely associated with PNI (26). While some studies have identified PNI as an independent risk factor for reduced survival, Garden et al. (3) suggested that only major nerve invasion significantly impacts prognosis, and that PNI was associated with progression to distant metastasis. In our study, both Cox regression and the Fine–Gray model confirmed the strong protective effect of the absence of PNI, although the Fine–Gray model yielded a more conservative estimate. These findings are consistent with clinical consensus: PNI is a core malignant feature of HNACC, and its absence indicates low tumor aggressiveness and a significantly more favorable survival prognosis.

Based on the proportional hazards model, we constructed a visual prognostic nomogram to estimate 3-year, 5-year, and 10-year CSS probabilities. Each category of the included variables was assigned a specific point value (marked by red circles), and the total prognostic score for an individual patient was calculated by summing the points for all variables, with a possible range of 0 to 409. The maximum total score of 409 corresponded to a 10-year CSS probability of 0.0114 (1.14%), representing the poorest prognostic profile. For an individual patient, the point values of all prognostic factors are summed to obtain the total score on the top scale. This total score is then projected vertically onto the 3-, 5-, and 10-year CSS probability scales at the bottom of the nomogram to derive individualized survival estimates. Notably, the prognostic factors exert additive effects on CSS. A higher total score corresponds to a shorter CSS and a greater risk of HNACC-related death. This optimized and user-friendly nomogram enables visual quantification of the contribution of each prognostic factor to CSS. Clinicians can directly map the total score to the corresponding probability scale at the bottom of the nomogram without the need for complex statistical calculations or specialized software.

The Fine-Gray model yielded a lower AIC value compared with the Cox model, demonstrating a superior model fit and supporting the application of the competing risks framework in this study. The C-index values exceeded 0.8, indicating strong discriminative performance. Calibration curves closely aligned with the reference line, confirming excellent agreement between predicted and observed outcomes. Results from the validation cohort also demonstrated the model’s stability, suggesting its potential utility for individualized risk assessment and clinical decision-making. For patients with a high total nomogram score, the model may support consideration of more aggressive individualized treatment strategies, such as intensive postoperative chemoradiotherapy and close multidisciplinary evaluation. For patients with lower scores, the model provides supportive evidence for the de-escalation of adjuvant therapy to avoid unnecessary treatment-related toxicity.

This study benefits from a large sample size derived from the high-quality SEER database and from the application of a competing-risks framework, which enhances the accuracy of prognostic estimation. Nevertheless, several limitations of this study should be acknowledged. First, the SEER database, which served as the source of our primary study cohort, consists predominantly of White patients, with smaller proportions of Black and other racial groups, whereas our external validation cohort comprised exclusively Asian patients from a single medical center in China. Although the nomogram demonstrated good predictive performance in the Asian cohort, the consistency of its prognostic value across diverse racial populations requires further confirmation in large-scale, multi-center, multiethnic prospective studies. Second, as a retrospective analysis, unmeasured confounding cannot be excluded. Potential misclassification of the cause of death in the SEER records could introduce information bias.

## 5 Conclusions

This study is the first to identify prognostic factors for CSS in nonmetastatic HNACC using a Fine–Gray competing risks model. Age ≥60 years, advanced T/N stage, omission of PORT, receipt of POCRT, and positive PNI were confirmed as independent adverse prognostic factors, with PNI and T and N stage being the most influential. The nomogram-based prognostic model developed in this study is a practical and easy-to-implement clinical scoring tool. It may assist clinicians in individualized prognostic assessment and evidence-based treatment decision-making for patients with nonmetastatic HNACC in routine practice, and it may also serve as a reliable instrument for risk stratification and follow-up management.

## Data Availability

The raw data supporting the conclusions of this article will be made available by the authors, without undue reservation.
