# Supplementary material for: A competing risk-based prognostic model for cancer-specific survival in non-metastatic head and neck adenoid cystic carcinoma
Source: Front Oncol. 2026 Mar 23;16:1752964. doi: 10.3389/fonc.2026.1752964 (PMC13050744; doi:10.3389/fonc.2026.1752964)
Supplement: Supplementary file 1 [file DataSheet1.pdf]

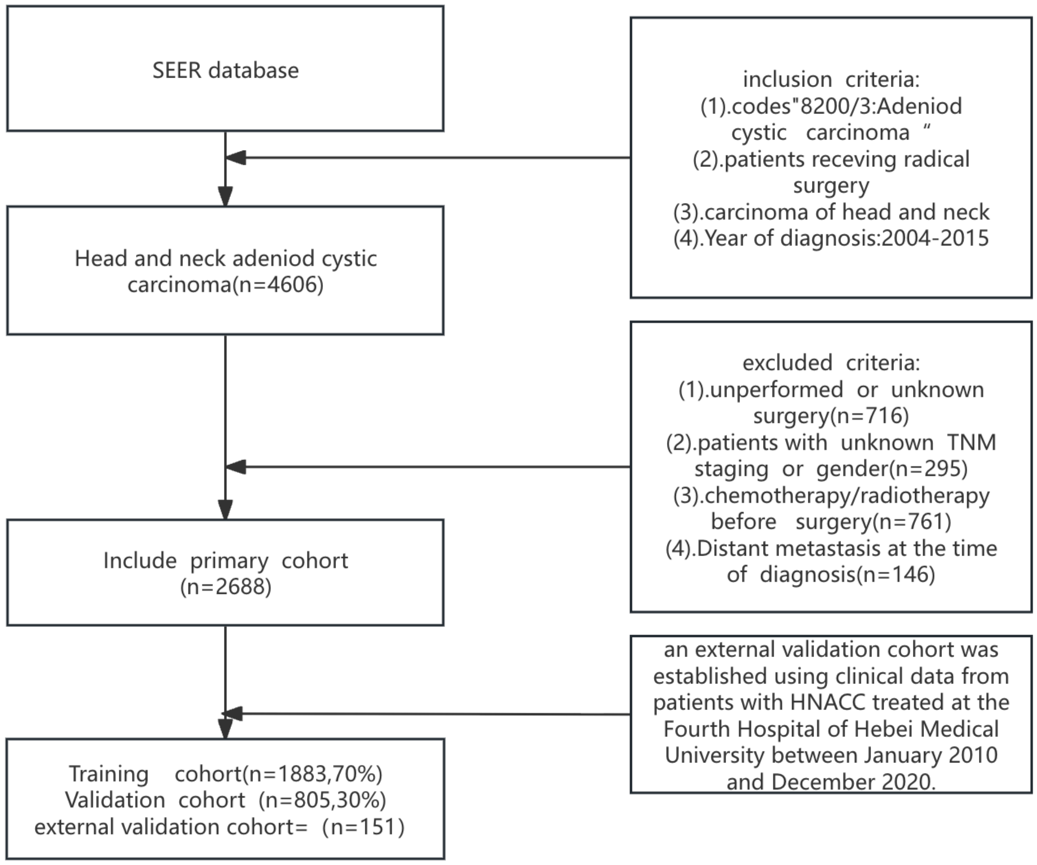

Supplementary Fig 1: Schematic illustration of the study design and patient-selection criteria

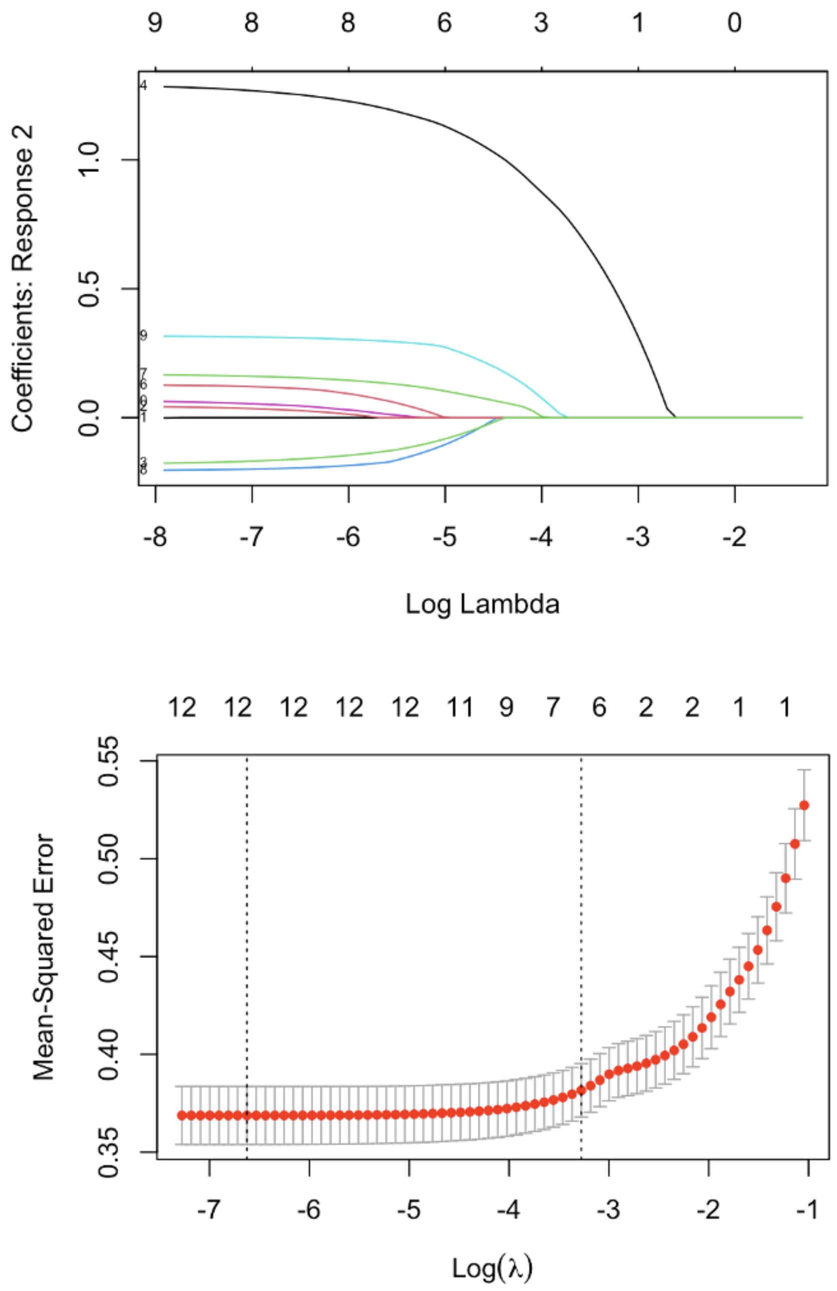

Supplementary Fig .2 Predictor screening using the least absolute shrinkage and selection operator (LASSO) regression with fivefold cross-validation.
